# Semi-automation of keratopathy visual acuity grading of corneal events in belantamab mafodotin clinical trials: clinical decision support software

**DOI:** 10.3389/fdgth.2023.1138453

**Published:** 2023-10-10

**Authors:** Mala K. Talekar, Jeffery L. Painter, Mica A. Elizalde, Michele Thomas, Heather K. Stein

**Affiliations:** ^1^ Oncology Clinical Development GSK, Collegeville, PA, United States; ^2^ Safety and Pharmacovigilance, GSK, Durham, NC, United States; ^3^ Regulatory Affairs, Precision Medicine and Digital Health, GSK, Rockville, MD, United States

**Keywords:** mobile app, clinical decision support tools, belantamab mafodotin, belamaf, belamaf eye examination, multiple myeloma, keratopathy

## Abstract

**Background:**

Belantamab mafodotin (belamaf) has demonstrated clinically meaningful antimyeloma activity in patients with heavily pretreated multiple myeloma. However, it is highly active against dividing cells, contributing to off-target adverse events, particularly ocular toxicity. Changes in best corrected visual acuity (BCVA) and corneal examination findings are routinely monitored to determine Keratopathy Visual Acuity (KVA) grade to inform belamaf dose modification.

**Objective:**

We aimed to develop a semiautomated mobile app to facilitate the grading of ocular events in clinical trials involving belamaf.

**Methods:**

The paper process was semiautomated by creating a library of finite-state automaton (FSA) models to represent all permutations of KVA grade changes from baseline BCVA readings. The transition states in the FSA models operated independently of eye measurement units (e.g., Snellen, logMAR, decimal) and provided a uniform approach to determining KVA grade changes. Together with the FSA, the complex decision tree for determining the grade change based on corneal examination findings was converted into logical statements for accurate and efficient overall KVA grade computation. First, a web-based user interface, conforming to clinical practice settings, was developed to simplify the input of key KVA grading criteria. Subsequently, a mobile app was developed that included additional guided steps to assist in clinical decision-making.

**Results:**

The app underwent a robust Good Clinical Practice validation process. Outcomes were reviewed by key stakeholders, our belamaf medical lead, and the systems integration team. The time to compute a patient's overall KVA grade using the Belamaf Eye Exam (BEE) app was reduced from a 20- to 30-min process to <1–2 min. The BEE app was well received, with most investigators surveyed selecting “satisfied” or “highly satisfied” for its accuracy and time efficiency.

**Conclusions:**

Our semiautomated approach provides for an accurate, simplified method of assessment of patients’ corneal status that reduces errors and quickly delivers information critical for potential belamaf dose modifications. The app is currently available on the Apple iOS and Android platforms for use by investigators of the DREAMM clinical trials, and its use could easily be extended to the clinic to support healthcare providers who need to make informed belamaf treatment decisions.

## 1. Introduction

Multiple myeloma (MM) is one of the most frequent hematological malignancies worldwide, with a median age at diagnosis of approximately 70 years (1). The median overall survival in patients with MM decreases with advancing disease stage. Survival is less than 7 years for those with Revised International Staging System stage II disease and 3.6 years for those with stage III disease (2). A typical course of MM includes relapses after initial treatment success and eventually the development of refractory disease, with the duration of response and progression-free survival (PFS) shortening with each successive line of therapy (3). A retrospective review of 7,261 patients with relapsed or refractory MM initiating first-line treatment between January 1, 2011, and May 31, 2017, revealed that median PFS and median overall survival varied considerably with subsequent lines of therapy. The median PFS declined from 12 months with first-line therapy to 3.5 months with fifth-line therapy. Additionally, median overall survival was 48.2 months with first-line therapy and 5.8 months with fifth-line therapy (4). Taken together, MM remains a challenging disease that is incurable for most patients, and the treatment of this patient population represents an unmet medical need (2).

Belantamab mafodotin (belamaf) is a B-cell maturation antigen–targeting antibody-drug conjugate that has demonstrated clinically meaningful antimyeloma activity in patients with heavily pretreated MM (5, 6). Belamaf was approved by the US Food and Drug Administration (FDA) for the treatment of patients with relapsed or refractory MM who have received at least 4 prior therapies, including an anti-CD38 monoclonal antibody, a proteasome inhibitor, and an immunomodulatory agent (7).

Belamaf treatment of patients with relapsed or refractory MM is being investigated in the DREAMM clinical trials. Results from DREAMM-1 demonstrated an overall response rate of 60% and a median PFS of 12 months (3.4 mg/kg); however, ocular toxicity was an emerging concern from DREAMM-1 (5, 8, 9). Corneal events occurred in 53% and 63% of treated patients for the dose-escalation and dose-expansion arms, respectively, and were more frequent with higher doses of belamaf (5, 8). Blurred vision was the most common reason for dose delays or dose reductions, yet most events (88%) were grade 1/2 and did not lead to treatment discontinuation (5). DREAMM-2 included a lower belamaf dose (2.5 mg/kg) and frequent ophthalmic examinations. Additionally, an ocular toxicity scale was developed to guide toxicity mitigation and management strategies for the DREAMM-2 trial (8). Corneal events occurred in 71% and 77% of patients treated with 2.5-mg/kg and 3.4-mg/kg belamaf, respectively. Most corneal events were corneal epithelium changes, and keratopathy was the most common reason for dose delays and dose reductions (6). Although not fully understood, several mechanisms have been proposed to explain belamaf-induced ocular toxicity (e.g., Fc receptor–mediated endocytosis, pinocytosis, and bystander toxicity) (10).

**Figure 1:**
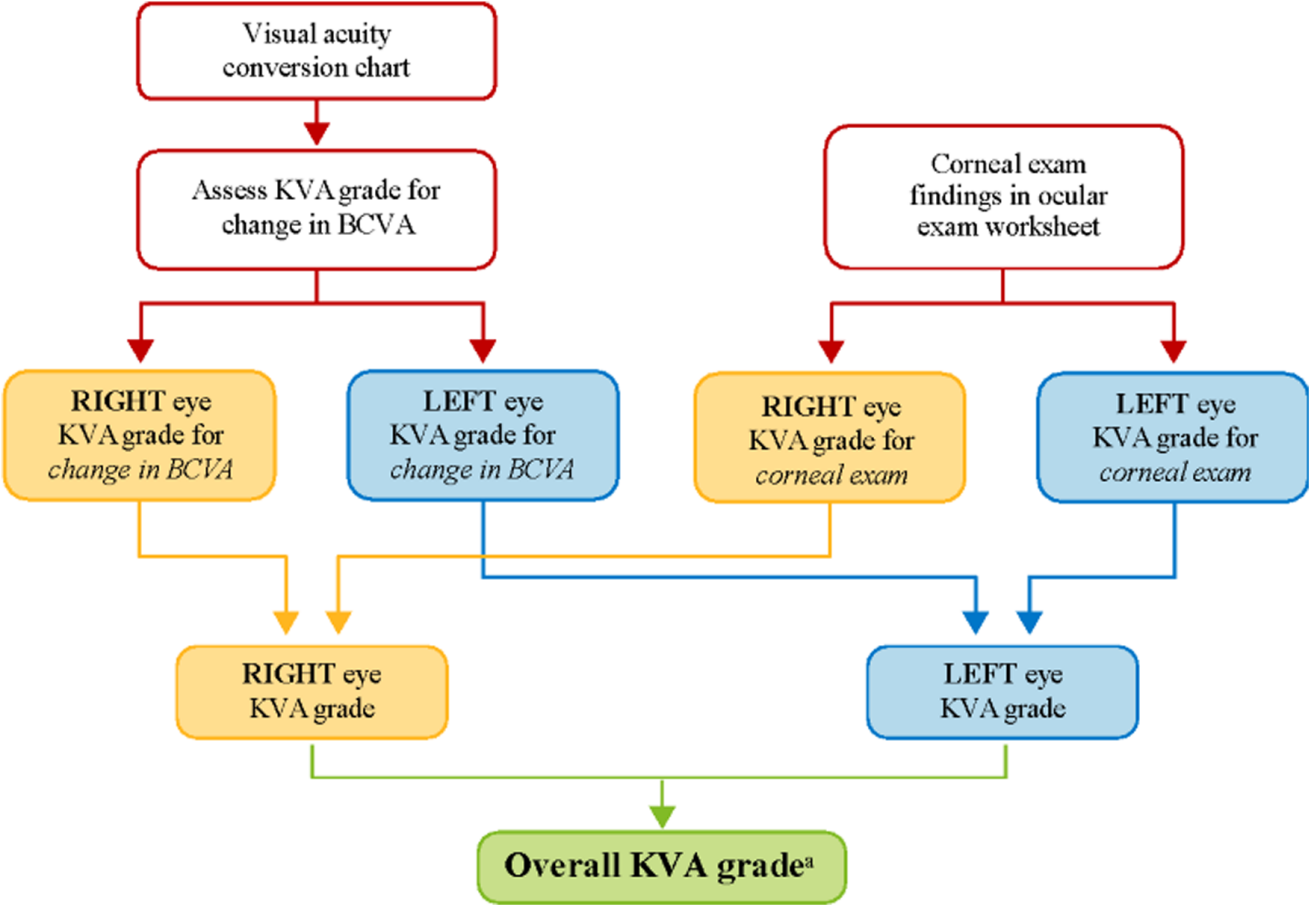
KVA grade determination using the KVA scale. **^a^**The developed semiautomated approach is being used at this step in the workflow. BCVA, best corrected visual acuity; KVA, keratopathy visual acuity.

Dose modifications (e.g., dose reductions and/or dose delays) of belamaf are effective in managing ocular symptoms and decreasing the severity of eye examination findings (2, 6); thus, patients can continue to derive the clinical benefit gained from belamaf without treatment discontinuation. To evaluate the need for dose modification, grading scales have been developed for determining the severity of belamaf-related corneal events. The grading scale for corneal events underwent refinement during the clinical development of belamaf as the database of corneal events expanded, mechanisms underlying the pathology of corneal events were proposed, and the reversibility of the events with dose modification became evident. For DREAMM clinical trials, dose modifications of belamaf are recommended based on a protocol-specified Keratopathy Visual Acuity (KVA) grading scale that incorporates corneal examination findings and the change in best corrected visual acuity (BCVA) from baseline. Dose modification of belamaf is based on an overall KVA grade that represents the most severe finding in two components of the eye examination (Figure 1). If the eyes differ in toxicity severity, dose modification is based on the eye with more severe toxicity.

**Figure 2:**
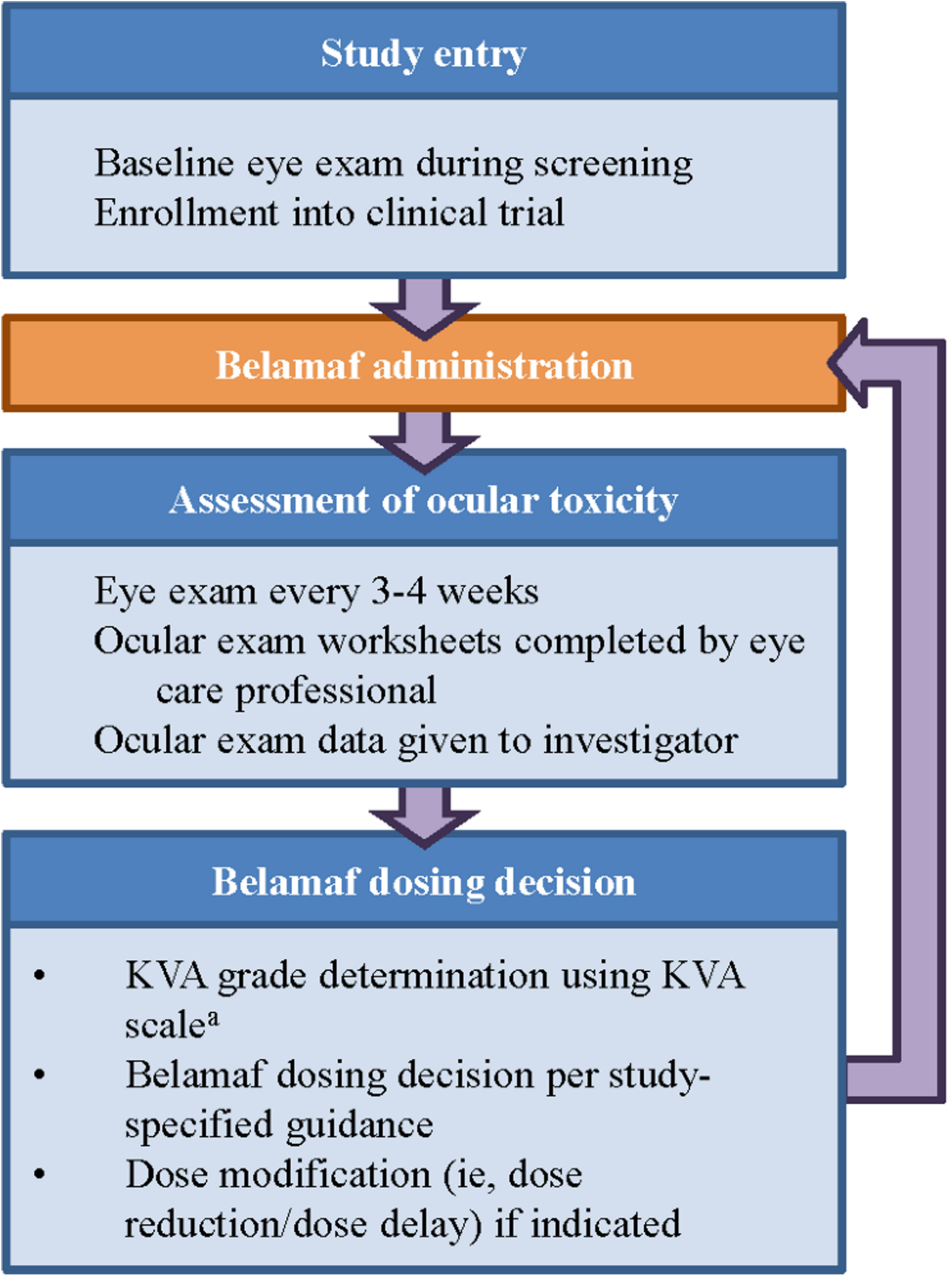
Workflow to determine dose modification of belamaf during clinical study. **^a^**The developed semiautomated approach is being used at this step in the workflow. KVA, keratopathy visual acuity.

The manual process of assessing the severity of corneal events using the KVA scale is complex, requiring coordinated steps between multiple care providers (e.g., eye care professional and the primary hematologist-oncologist). Collecting visual acuity data points from the baseline and current eye examination (e.g., on the ocular examination worksheet), performing unit conversions for visual acuity [e.g., converting from logarithm of the minimal angle of resolution (logMAR) to Snellen], and understanding the patient's clinical status all contribute to the cognitive load experienced by investigators when determining the proper dose modification for belamaf. The computation of the overall KVA grade is just one part of the entire process necessary for the investigator to make a dosing recommendation, but it is a critical piece in assessing the progress of the patient and their treatment journey (Figure 2).

The limitations associated with the traditional paper process of computing KVA grades and its impact on proper investigator-determined dose modification of belamaf provided the driving force to create a semiautomated method for the accurate computation of KVA grades. A tool that could assist investigators with appropriate dosing recommendations while minimizing the time necessary to reach that determination would be beneficial in research studies and in clinical settings. Thus, our team developed a streamlined system that would take the data points entered by the investigator for the visual acuity examination and the corneal examination findings and furnish an overall KVA grade in less than a minute. In addition, the system was designed to limit the data inputs to only those that are valid to prevent the documentation of logical errors. We demonstrate that our protocol to semi-automate this process using finite state machines and application logic has the potential to reduce the number of errors observed when determining patient dosing in ongoing clinical trials by our in-stream medical data safety review teams. Finally, we show how the manual process was transformed from prototype to a mobile app, the Belamaf Eye Exam (BEE) app, now available for download and use by trained clinical investigators^1^.

## 2. Methods

To have a unified approach to the assessment of corneal events, the DREAMM clinical trials provide participating clinical sites and investigators with guidance documents to enable accurate determination of the KVA grades. To assess the change in BCVA, the investigator is provided with a visual acuity conversion chart that permits the interconversion of different units of visual acuity (Supplementary Table S1). The investigator is also provided with guidance for assessing and interpreting changes in lines of vision between the baseline and current eye examination and for determining the KVA grade for that change (Supplementary Table S2) and a KVA scale that provides guidance on assigning a KVA grade to each of the components of the eye examination (Supplementary Table S3).

### 2.1. Step 1: compute KVA grade for left and right eye

The first step is to compute the grade change in visual acuity from baseline based on the current visit reading. For example, a baseline BCVA of 20/20 (Snellen) that changes to 20/25 would be classified as KVA grade 1 (Supplementary Table S2). However, the KVA grade scale demonstrates that a change from one reading to another cannot be simplified into merely counting the number of lines between the two. Indeed, the line change in visual acuity is not always accurately captured by the selected KVA grade when using the paper method.

Thus, one aspect of the app design was the translation of the change in BCVA from baseline to current values into a finite-state machine data representation. A finite-state machine, or finite-state automaton (FSA), is a mathematical model of computation commonly used to perform a predetermined set of actions based on a presented sequence of events (11). Thus, the change in BCVA for a patient's eyes represents an ideal application of this principle, whereby a common calculation could be automated. For simplicity, we abstracted this process independent of the left or right eye and applied the FSA model to acquire the KVA grade for the change in BCVA for each eye.

Converting the tables into an FSA representation was initially done in the JavaScript programming language. Using a JavaScript object notation (JSON) object as our data model, we defined the transition states for all possible baseline values and all possible outcomes. “JSON is a native data form for JavaScript, which means no special Application Programming Interfaces (APIs) or jars are needed to process JSON data. These features make JSON an ideal format for data exchanging in web service applications (12).” Each state is represented as a direct map in the user interface such that the transition from any baseline value is mapped to an individual grade (i.e., grade 1–3, or 4).

**Figure 3:**
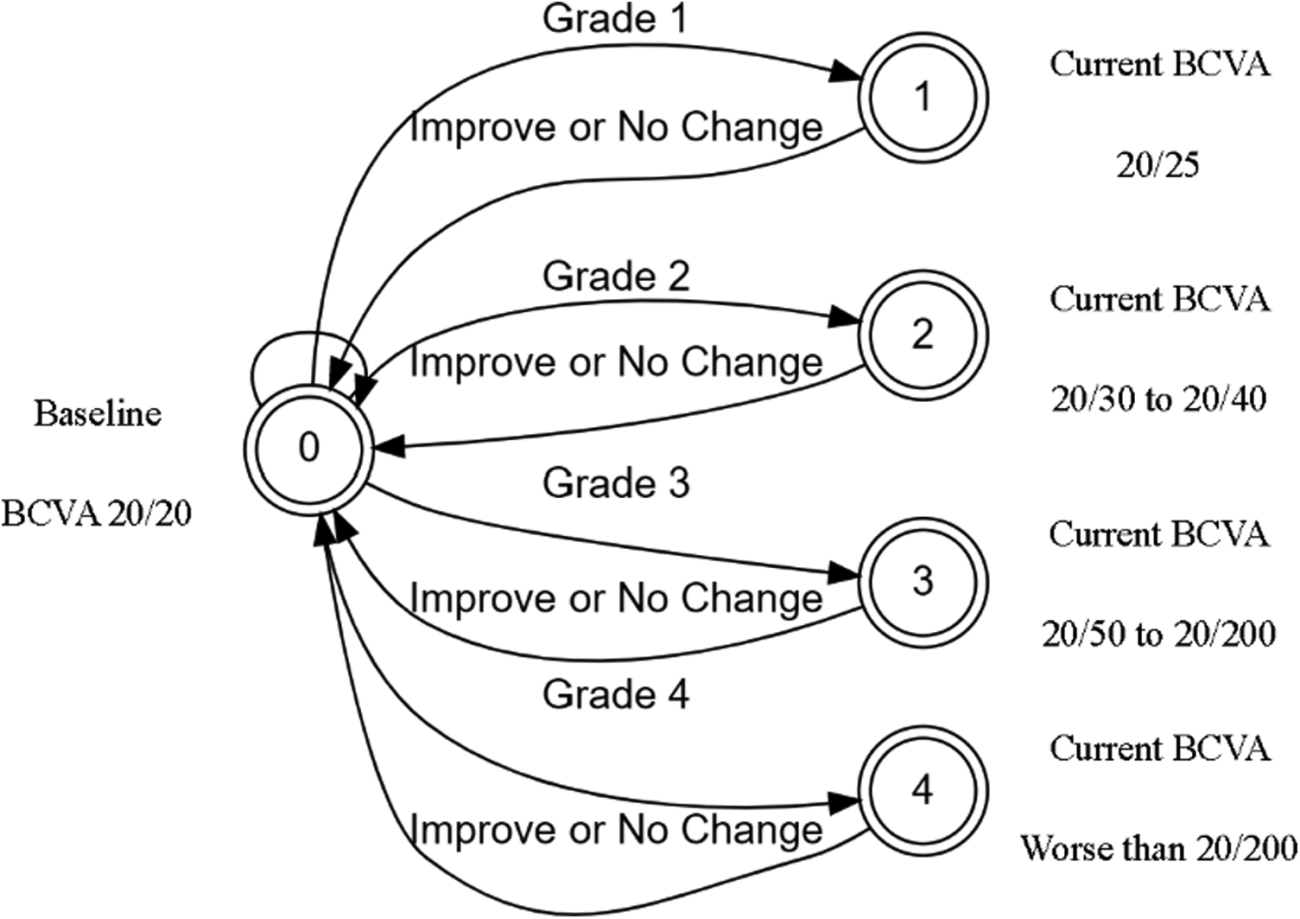
BEE app FSA model for computing change in BCVA of 20/20. BCVA, best corrected visual acuity; FSA, finite-state automaton.

**Figure 4:**
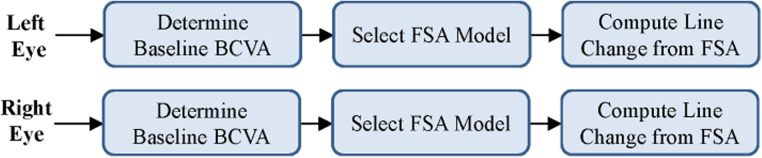
Selection of appropriate FSA model based on user input. BCVA, best corrected visual acuity; FSA, finite-state automaton.

The initial prototype was developed as a web application for agile testing and development. The application used only JavaScript and HTML to build an interactive tool that could be used to test various patient scenarios and KVA calculations using the FSA model. The developed FSA model assumes that any improvement or no change in BCVA has a KVA grade of “not applicable (N/A)” since stable or improving BCVA has no impact on dosing recommendations. Since each state transition is clearly defined and unambiguous, we were able to use the process described by Press (13) to convert entries in Supplementary Table S2 into a limited-entry, nonambiguous table format for use in our application. The way the FSA model handles the “N/A” categories is to exclude the jump from one state to another. An example of a complete FSA demonstrating the grade change with a baseline BCVA score of 20/20 (via Snellen) is provided (Figure 3). If the current BCVA is better or has not changed, the computed grade change is zero and reported in the user interface as “N/A.” Depending on the current baseline readings, the FSA is applied and the line change for each eye is computed. The result of these efforts was a proof-of-concept, semiautomated KVA grading tool that contains over 20 distinct FSA models for all other baseline values. A high-level diagram illustrates how the FSA model is selected and applied to each eye (Figure 4). Depending on baseline BCVA, the appropriate FSA model is selected and applied to the right and left eyes to compute the grade change score for each.

An additional benefit of using FSA models is that they have been designed independently of the units of measurement (e.g., Snellen, logMAR, decimal). Therefore, we only had to demonstrate that the transition table was correct for a single value, and the user interface automatically adjusted to the proper unit conversions based on the user's preference for data input. This automation is absent from the paper process, relying on the user to correctly convert all of their data points into a common unit (e.g., Snellen) before assigning a KVA grade using the KVA grade scale (Supplementary Table S3).

### 2.2. Step 2: compute grade based on corneal exam

Despite being computable by a program, the corneal examination findings are even more difficult for the investigator to interpret if they are not intimately familiar with the process. A single question can change the entire patient outcome and could easily be missed if the overall KVA grade for the patient is not carefully computed for either eye. With automation, this complex step can be converted into a simple set of multiple choice, dropdown-driven questions for the user. The logic is embedded within the application through a set of conditional “if/then” statements that determine the grade based on the provided guidance statements (Supplementary Table S3). By applying various application logic and rules, the system can quickly eliminate answers that are impossible scenarios, which a simple paper form would not be able to accommodate.

For the DREAMM studies using the KVA grading scale, there are 5 questions related to the corneal examination, with multiple outcomes for some questions. When prompted about the existence of a superficial punctate keratopathy, the user responds with “none,” “mild,” “moderate,” or “severe.” In a modified version of the BEE app running for an additional clinical trial, one prompt asks whether the patient has a “clear cornea.” If the answer is “Yes,” then all subsequent questions about the corneal examination are unnecessary to answer since this choice automatically assigns the grade in question to the lowest level possible (i.e., grade 0). Thus, the application logic, response presentation, and elimination of unreasonable outcomes makes the semiautomated process more time efficient for the investigator.

### 2.3. Step 3: compute overall KVA grade

Once the KVA grade change for both eyes has been computed and the logic applied to determine the grade based on the corneal examination, the final step is to abstract and combine these data and divide the grading process into a functional programming model to compute the overall KVA grade. A pseudoalgorithm of this process uses the change in BCVA grade and the corneal examination finding grade for the left and right eyes to compute the overall KVA grade.

### 2.4. Implementation of models into user interface design

We used a data-driven approach when developing the prototype user interface (UI) design (14). The manual process was broken down into the minimal, discrete steps necessary to compute the overall KVA grade. The UI was also designed to conform to the standards developed from ophthalmologist findings (e.g., right eye findings are typically shown on the left-hand side of the screen rather than the right side since from the eye care professional's point of view, the patient's right eye is on the eye care professional's left).

**Figure 5:**
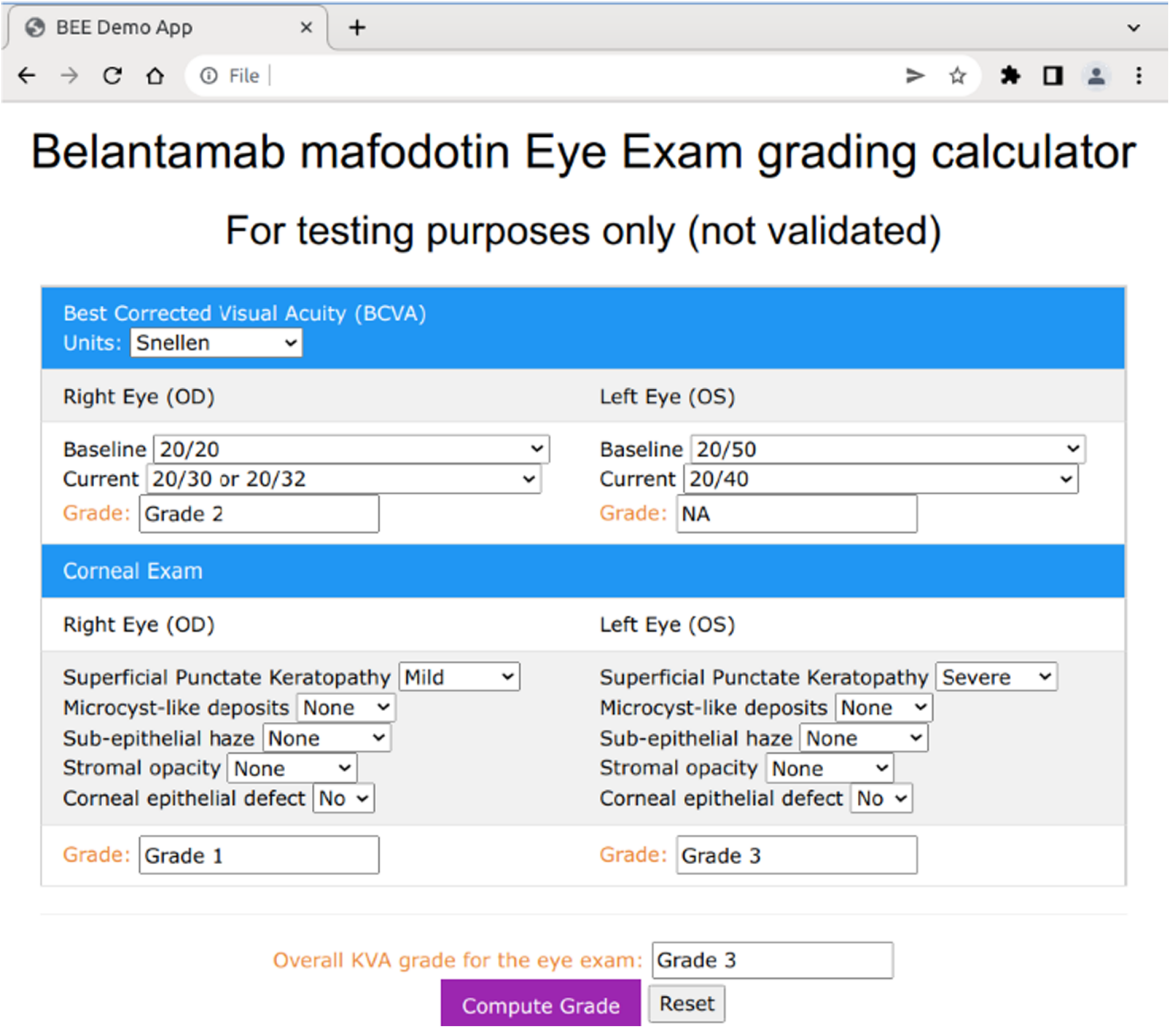
Web-based BEE app user interface design. BCVA, best corrected visual acuity.

The BCVA computation only requires that the user select the baseline and current readings for visual acuity for both the left and right eyes from the dropdown menu (Figure 5). The corneal examination findings follow a similar procedure, in which the user selects the appropriate answer to each of the questions of interest (e.g., superficial punctate keratopathy) for the current eye examination. Below the right and left eye in each section, a grade box is presented that will show the computed grade for each of the subcomponents included in the grade determination (Figure 5). The user then clicks on the “Compute Grade” button to generate the overall KVA grade.

In the mobile BEE app, this process has been further simplified through a series of guided steps, along with haptic touch/response buttons to accommodate data entry on a mobile device, but the underlying logic and algorithms used to compute the overall KVA grade remain the same.

### 2.5. From prototype to mobile app

A prototype application demonstrated functional capabilities that could enable faster computation of the overall KVA grade and reduce errors by adding the FSA model and application logic. The GSK internal mobile app development team further refined this prototype into a mobile app that could be deployed in the field and used by investigators. Indeed, the construction of the initial version of the application in a pure JavaScript implementation facilitated the conversion of the prototype into a real-world mobile app. The development team initially focused on modifying the application to run on the Apple iOS platform. The team worked in an agile process, iterating through sprints and software builds, to develop and test the mobile UI with internal subject matter experts. Working with a cross-functional team of clinical, software, and safety experts, the publication of the app took approximately 6 months from the initial conception. Representative screenshots from the mobile app that demonstrate the inputs necessary to determine an overall KVA grade are shown in the Supplementary Figures.

## 3. Results

### 3.1. System testing and performance of the mobile BEE app

Prior to deployment, the app underwent a robust Good Clinical Practice (GCP) validation testing process in alignment with GSK standard operating procedures, and the outcomes and deliverables were reviewed by medical personnel and systems integration teams. Because the BEE app is intended to support activities performed during the conduct of a clinical trial, the BEE app is subject to GCP. GCP validation for computer systems in GSK involves “…demonstrating through documented evidence (e.g., paper-based, electronic record, or hybrid) that a system is reliable, fit for its specified purpose, and compliant with regulatory and GSK requirements.” It is important to note that validation is an ongoing process that will cover the entire BEE app life cycle from specifying, developing, testing, implementing, operating, and managing (e.g., enhancements, change requests) through decommissioning.

GSK has adopted a risk-based approach to validation, including testing, and we used a predefined, GSK-approved validation methodology containing procedures for planning, conducting, and reporting validation. System requirements (50 for Android version of BEE App, 26 for Apple) spanning functionality, security, UI/UX, data, operational and compliance categories, were implemented and tested in order to support 26 total user requirements. Seven end-users executed 6 test cases resulting in detection, correction, re-testing and closure of 9 total defects (3 for Apple, 6 for Android) via 2 cycles of validation testing, including regression testing by an independent tester. Upon successful completion of the requirements of GCP validation (38 approved validation documents in total), the BEE app was released and made available for use via the Apple iOS Store on April 13, 2022, for iOS devices and was most recently published on the Google Play Store for Android devices on July 7, 2022. The BEE app is now supported by over 18,000 unique smartphone device models worldwide (≈30 countries). Initial results based on use of the iOS version of BEE app have demonstrated that the time to compute a patient's overall KVA grade can be reduced from a 20- to 30-min paper process to less than 1–2 min using our semiautomated KVA computation framework.

### 3.2. Investigator feedback on BEE app use and satisfaction

GSK provided initial users (*N* = 6) of the BEE app with the opportunity to record their usage patterns and satisfaction through an online survey. Based on the responses collected to date (50% response rate, *N* = 3), most investigators used the BEE app “1–5 times a week” or when uncertain about a KVA grading. Favorable aspects of the BEE app were increased accuracy (1/3), time savings (1/3) and app is quick/good performance (2/3); All survey respondents as of December 2022 have rated their satisfaction with the App as “satisfied” or “highly satisfied.”

### 3.3. Regulatory perspective on software as medical device

Software applications are increasingly being developed and then used by healthcare professionals, caretakers, and patients for a variety of healthcare uses. These software solutions are known as clinical decision support (CDS) software. CDS tools have a number of benefits that can promote patient safety (15), which have led the FDA and EU to publish guidance documents regarding their classification for safe and effective use. The FDA describes CDS as “a variety of tools including, but not limited to computerized alerts and reminders for providers and patients, clinical guidelines, condition-specific order sets, focused patient data reports and summaries, documentation templates, diagnostic support, and contextually relevant reference information” (16). According to FDA and EU guidance documents, the BEE app would not be within the purview of medical device regulations (Supplementary Appendix and Tables S4–S6).

## 4. Discussion

The off-target corneal events associated with belamaf treatment in patients with MM may be mitigated with accurate eye assessments and prompt modification of dosing (i.e., dose delay or reduction) (2, 6). However, these processes require the concerted effort between eye care specialists and study investigators and can be impacted by a time-consuming and error-prone manual calculation method. The development of the BEE app was driven by a need to provide a user-friendly, “calculator-like tool” for study investigators completing ocular toxicity assessments. The semiautomated approach provides for an accurate, simplified method of patient assessment that reduces the number of potential errors and more quickly delivers information critical for belamaf dose modifications. Moreover, using an FSA model, the application is capable of transitioning between user inputs and reliable calculations, including the ability to accept a variety of measurement units (i.e., Snellen, decimal, or logMAR). Guided steps in the mobile app may also provide clinical decision support for healthcare providers.

Despite the benefits of the BEE app, determination of KVA grade still requires accurate data entry by the user (e.g., investigator or healthcare provider). Additionally, in its current state, the mobile app does not save data entry and does not provide dosing modification recommendations. The decision to modify a belamaf dose requires the investigator to take into consideration their study-specific guidance, a patient's clinical status, other nonocular toxicities (if present), and their professional interpretation of the overall KVA grade.

## 5. Conclusion

Belamaf treatment-related corneal events are common but may be adequately managed by close liaison with eye care professionals and following the KVA scale guidelines (17). This report describes the development and testing of a semiautomated application for computing the overall KVA grade that is accurate and more time efficient compared with the traditional paper procedure. DREAMM clinical trial investigators should follow their study-specific guidance to interpret the KVA grade outputs from the BEE application and to determine whether a dose modification is required for belamaf.

## Data Availability

The original contributions presented in the study are included in the article/**Supplementary Material**, further inquiries can be directed to the corresponding author.

## Footnotes

^1^Note that this application was designed for medically trained staff to assist in clinical decision making. It is not an application intended for direct use by patients.
