# Supplementary material for: Semi-automation of keratopathy visual acuity grading of corneal events in belantamab mafodotin clinical trials: clinical decision support software
Source: Front Digit Health. 2023 Oct 10;5:1138453. doi: 10.3389/fdgth.2023.1138453 (PMC10597720; doi:10.3389/fdgth.2023.1138453)
Supplement: Supplementary file 1 [file Datasheet1.pdf]

Supplementary Material

Semi-Automation of Keratopathy Visual Acuity Grading of Corneal Events in Belantamab Mafodotin Clinical Trials: Clinical Decision Support Software

Mala K. Talekar, MBBS[1], Jeffery L. Painter, JD[2], Mica A. Elizalde, RAC[3], Michele Thomas, BS, MBA[1], Heather K. Stein, MD[1]

[1]GSK, Collegeville, PA, United States

[2]GSK, Durham, NC, United States

[3]GSK, Rockville, MD, United States

*** Correspondence:**
Corresponding Author
drmala.kt@gmail.com

1 Supplementary Text

The FDA regulates any software as a medical device if it is used to provide decision support for the diagnosis, treatment, prevention, cure, or mitigation of diseases or conditions. A draft guidance document, *Clinical Decision Support (CDS) Software*, describes their regulatory approach to CDS software functions and includes recent changes to section 3060(a) of the Cures Act amended section 520(o) of the Food Drug and Cosmetic (FD&C) Act, which exclude certain software functions from the definition of a device (1, 2). Software must meet 4 criteria to be excluded from the definition of a medical device (Supplementary Table 4). Based on the use of the application and the requirements in the amended section 520(o) of the FD&C Act, the BEE app is considered a nondevice CDS software by the FDA.

The EU defines medical devices under regulation 2017/745 of 2017 (3). According to this regulation, software is considered a medical device if it is to be used, alone or in combination, for human beings for one or more of the following specific medical purposes: diagnosis, prevention, monitoring, prediction, prognosis, treatment, or alleviation of disease; diagnosis, monitoring, treatment, alleviation of, or compensation for, an injury or disability; investigation, replacement, or modification of the anatomy or a physiological or pathological process or state; and/or providing information by means of in vitro examination of specimens derived from the human body, including organ, blood, and tissue donations.

Rule 11 of Annex VIII in EU regulation 2017/745 classifies medical device software in the EU (Supplementary Table 5). According to this rule, the BEE app would be classified as a class IIa medical device; however, the Medical Device Coordination Group (MDCG) has published additional guidance: MDCG 2019-11, *Guidance on Qualification and Classification of Software in Regulation*

(EU) 2017/745—Medical Device Regulation (MDR) and Regulation (EU) 2017/746—IVDR, which states that not all software used within health care is qualified as a medical device, including software that serves a medical purpose (4). Indeed, the guidance explains that software performing a simple search, such as the retrieval of records by matching record metadata against record search criteria, does not qualify as a medical device.

Additionally, the EU *Manual on Borderline and Classification in the Community Regulatory Framework for Medical Devices*, version 1.22 provides examples of software, medical devices, and in vitro diagnostic medical devices that do not meet the definition of a device (Supplementary Table 6) (5). The BEE app is not processing, analyzing, creating, or modifying information or data. The software searches a BCVA KVA table and corneal examination KVA table and returns the appropriate KVA grade based on the values input by the healthcare professional. Thus, the BEE app does not fulfil the definition of a medical device.

2 Supplementary Figures and Tables

2.1 Supplementary Figures

Supplementary Figure 1. BEE App screens 1 and 2 for the input of BCVA for the left and right eyes

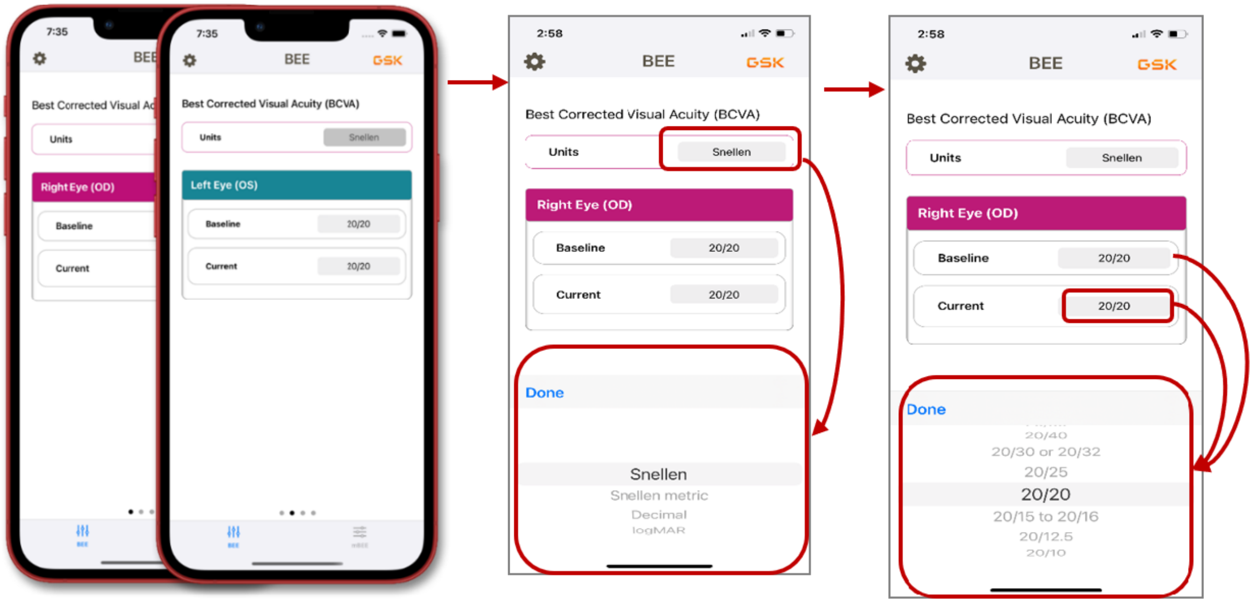

Supplementary Figure 1. The BCVA for the left and right eyes is entered on the first 2 screens of the BEE app for both the baseline examination and the current examination. The investigator can choose which unit measure to use for examination findings for BCVA (eg, “Snellen metric,” “Decimal,” or “logMAR”) and enter “Baseline” and “Current” values.

BCVA, best corrected visual acuity.

Supplementary Figure 2. BEE App screens 3 and 4 for the input of findings from the current corneal examination

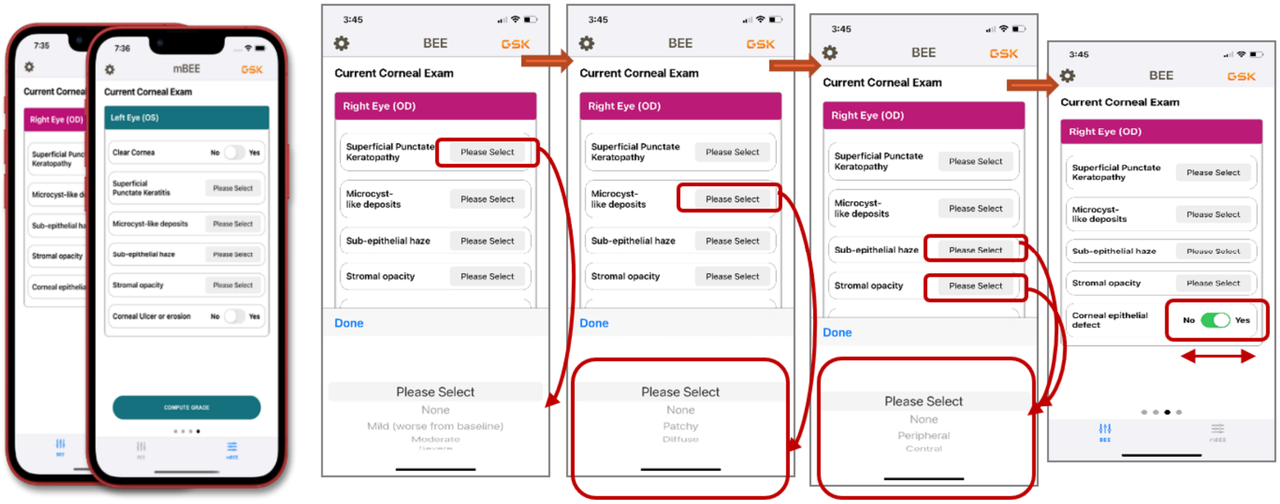

Supplementary Figure 2. The investigator is prompted to add corneal examination findings for both the left and right eyes on these BEE app screens. The investigator must respond to each of 5 questions regarding the current eye examination including “superficial punctate keratopathy,” “microcyst-like deposits,” “subepithelial haze,” “stromal opacity,” and “corneal epithelial defect.” The investigator can choose their response from the dropdown menus for each entry. Once the entry is completed, the investigator will tap a button at the bottom of the screen labeled “Compute grade,” which will take them to the final “Grading” screen.

Supplementary Figure 3. BEE App screen 5 for the computation of KVA grade based on user inputs

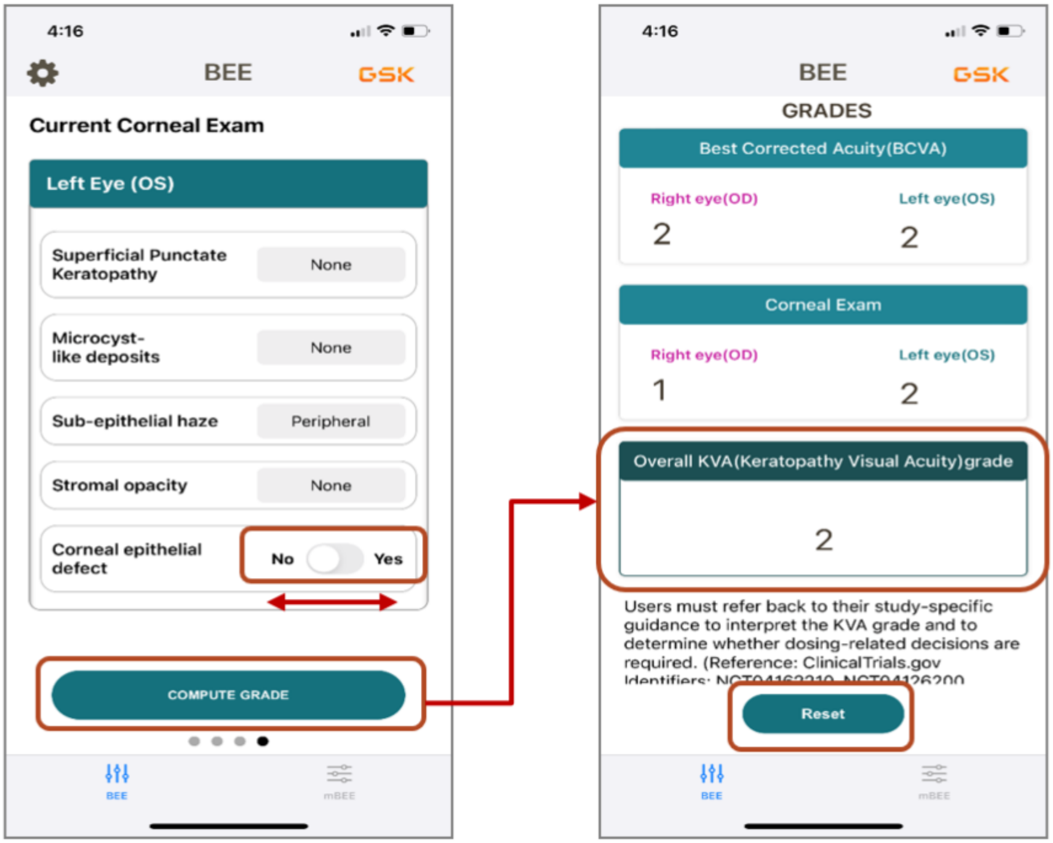

Supplementary Figure 3. On the final "Grades" screen, the investigator is provided with the KVA grade(s) for change in BCVA and for the corneal examination for both the right and left eyes as determined by the BEE app. Additionally, the highest (worst) grade from each of the individual components (ie, BCVA left eye, BCVA right eye, corneal examination left eye, and corneal examination right eye) is output as the overall KVA grade for the eye examination. The investigator is prompted to refer to their study specified guidance at the bottom of the screen. The tool can be reset in preparation for a new assessment by tapping the "Reset" button.

BCVA, best corrected visual acuity; KVA, keratopathy visual acuity.

2.2 Supplementary Tables

Supplementary Table 1. Visual acuity conversion chart

Snellen	Snellen (metric)	logMAR	Decimal
No equivalent value[a]			
20/20000 (18)[b]	6/6000	3.0	0.001
20/2000 (18)[c]	6/600	2.0	0.010
20/400	6/120	1.3	0.05
20/320	6/96	1.2	0.063
20/250	6/76	1.1	0.08
20/200	6/60	1.0	0.10
20/160	6/48	0.9	0.125
20/150			
20/125	6/38	0.8	0.15-0.16
20/100	6/30	0.7	0.20
20/80	6/24	0.6	0.25
20/70			
20/63	6/20	0.5	0.3-0.32
20/60			
20/50	6/15	0.4	0.40
20/40	6/12	0.3	0.50
20/32	6/10	0.2	0.6-0.63

20/30			0.67-0.7
20/25	6/7.5	0.1	0.80-0.9
20/20	6/6	0.0	1.00
20/16	6/5	−0.1	1.2-1.25
20/12.5	6/3.8	−0.2	1.5-1.6
20/10	6/3.0	−0.3	2.0

[a] Non-Snellen acuity of "light perception" or "no light perception" do not have corresponding values.
[b] Hand motion at 2 feet.
[c] Counting fingers at 2 feet.

Supplementary Table 2. Guidance for assessing changes in best corrected visual acuity for KVA grading

**Baseline Best Corrected Visual Acuity (BCVA)**[a]	**Grade 1** (1 line decrease from baseline BCVA)	**Grade 2** (2-3 lines decrease from baseline BCVA)	**Grade 3** (>3 lines decrease from baseline BCVA but not worse than 20/200)	**Grade 4** (BCVA worse than 20/200)
20/10	20/12.5	20/15 to 20/16 or 20/20	20/25 to 20/200	Worse than 20/200
20/12.5	20/15 to 20/16	20/20 to 20/25	20/30 to 20/200	Worse than 20/200
20/15 to 20/16	20/20	20/25 to 20/30 or 20/32	20/40 to 20/200	Worse than 20/200

20/20	20/25	20/30 to 20/40	20/50 to 20/200	Worse than 20/200
20/25	20/30 or 20/32	20/40 to 20/50	20/60 to 20/200	Worse than 20/200
20/30 to 20/32	20/40	20/50 to 20/60 or 20/63	20/70 to 20/200	Worse than 20/200
20/40	20/50	20/60 to 20/70 or 20/80	20/100 to 20/200	Worse than 20/200
20/50	20/60 or 20/63	20/70 to 20/100	20/125 to 20/200	Worse than 20/200
20/60 to 20/63	20/70 or 20/80	20/100 to 20/125	20/150 to 20/200	Worse than 20/200
20/70 to 20/80	20/100	20/125 to 20/150 or 20/160	20/200	Worse than 20/200
20/100	20/125	20/150 to 20/160 or 20/200	N/A	Worse than 20/200
20/125	20/150 or 20/160	20/200	N/A	Worse than 20/200
20/150 to 20/160	20/200	N/A	N/A	Worse than 20/200

Worse than 20/160	N/A	N/A	N/A	Any further reduction from baseline is considered grade 4

[a] BCVA presented as Snellen equivalent.

BCVA, best corrected visual acuity.

Supplementary Table 3. Keratopathy visual acuity (KVA) scale

		Grade per KVA Scale			
		**Grade 1**	**Grade 2**	**Grade 3**	**Grade 4**
**Corneal Toxicities[a]**	**Corneal examination finding(s)**	**Mild superficial keratopathy**<br><br>*Mild superficial punctate keratopathy (documented worsening from baseline), with or without symptoms*	**Moderate superficial keratopathy**<br><br>*Any/or a combination of:*<br>• **Moderate** superficial punctate keratopathy<br>• **Patchy** microcyst-like deposits<br>• **Peripheral** subepithelial haze<br>• **New peripheral** stromal opacity	**Severe superficial keratopathy**<br><br>*Any/or a combination of:*<br>• **Severe** superficial punctate keratopathy<br>• **Diffuse** microcyst-like deposits **involving the central cornea**<br>• **Central** subepithelial haze<br>• **New central** stromal opacity	**Corneal epithelial defect**<br><br>*Such as* ***corneal erosion(s)*** *or* ***corneal ulcer(s)***[b]

	**Change in BCVA[c]**	Decline from baseline of **1 line** on Snellen Visual Acuity	Decline from baseline of **2-3 lines** (and Snellen Visual Acuity not worse than 20/200)	Decline from baseline by **>3 lines** (and Snellen Visual Acuity not worse than 20/200)	Snellen Visual Acuity **worse than 20/200**

[a] Dose modification should be based on the most severe finding. If eyes differ in severity, dose modification guideline should be applied based on the more severe eye.
[b] Corneal ulcer by definition means an epithelial defect with underlying stromal infiltration.
[c] Changes in visual acuity due to ***treatment-related*** corneal findings.

- For participants who have BCVA worse than 20/20 in either eye at baseline, dose modification for that eye will be determined by the worsening of vision from baseline only (not by absolute BCVA at the visits).
- If a participant has a baseline BCVA of 20/200 or worse in an eye, then belantamab mafodotin–related changes in vision in the other eye will drive the dose modification. If a participant has baseline BCVA of 20/200 or worse in both eyes, then the decision to delay or reduce belantamab mafodotin dose will be based on principal investigator’s assessment of benefit vs risk based on corneal examination findings following a discussion with a qualified eye care specialist.

BCVA, best corrected visual acuity; KVA, keratopathy visual acuity.

Supplementary Table 4. FDA nondevice CDS software requirements

**Nondevice CDS Criteria**	**Criteria Fulfilled by BEE App**
Not intended to acquire, process, or analyze a medical image or a signal from an in vitro diagnostic (IVD) device or a pattern or signal from a signal acquisition system (section 520(o)(1)I of the FD&C Act)	Yes. The BEE app does not acquire, process, or analyze a medical image or signal for an IVD or other system.
Intended for the purpose of displaying, analyzing, or printing medical information about a patient or other medical information (such as peer-reviewed clinical studies and clinical practice guidelines; section 520(o)(1)I(i) of the FD&C Act)	Yes. The BEE app displays medical information about a patient and displays information based on the Blenrep approved drug label.
Intended for the purpose of supporting or providing recommendations to a healthcare professional about prevention, diagnosis, or treatment of a disease or condition (section 520(o)(1)I(ii) of the FD&C Act)	Yes. The BEE app is used to support a healthcare professional by providing a KVA grade.
Intended for the purpose of enabling such healthcare professional to independently review the basis for such recommendations that such software presents so that it is not the intent that such healthcare professional rely primarily on any of such recommendations to make a clinical diagnosis or treatment decision regarding an individual patient (section 520(o)(1)I(iii) of the FD&C Act)	Yes. The BEE app uses BCVA KVA tables and corneal examination KVA tables that are made available to the healthcare provider through the Blenrep approved drug label, Blenrep investigator’s brochure and/or belamaf clinical trial protocol(s). The healthcare professional can use the information provided to them through one of the options above to manually determine or verify the KVA grade.

BCVA, best corrected visual acuity; CDS, clinical decision support; FDA, Food and Drug Administration; FD&C, Federal Food, Drug & Cosmetic; IVD, in vitro diagnostic; KVA, keratopathy visual acuity.

Supplementary Table 5. MDR 2017/745 software classification rule 11

MDR Annex VIII, Rule 11	Applicability to BEE App
Software intended to provide information that is used to make decisions for diagnosis or therapeutic purposes is classified as class IIa.	Yes. The BEE app will be used to support an HCP in the determination of the patient's KVA grade.
Except if such decisions have an impact that may cause death or an irreversible deterioration of a person's state of health, in which case it is in class III.	No. The results from the BEE app are unlikely to cause death or irreversible deterioration of health, and it is therefore not likely to be considered a class III device.
Or a serious deterioration of a person's state of health or a surgical intervention, in which case it is classified as class IIb.	No. The BEE app will be used to support an HCP in the determination of the patient's KVA grade. KVA is not a serious determination of a person's state of health and does not require a surgical intervention.
Software intended to monitor physiological processes is classified as class IIa, except if it is intended for monitoring of vital physiological parameters, where the nature of variations of those parameters is such that it could result in immediate danger to the patient, in which case it is classified as class IIb.	No. The BEE App does not monitor physiological parameters. It searches a BCVA KVA table and corneal examination KVA table and returns the appropriate grade based on the values input by the HCP.
All other software are classified as class I.	No. The BEE app meets one of the items above.

BCVA, best corrected visual acuity; HCP, healthcare provider; KVA, keratopathy visual acuity, MDR, Medical Device Regulation.

Supplementary Table 6. Example 9.4 from the EU manual on borderline medical devices

**Background**	The product in question consists of a software application that allows for faster consulting/reading of an international guideline regarding the Classification of Malignant Tumours (TNM) issued by the UICC (International Union Against Cancer) in the view of classification of cancer by anatomic disease extent.<br>Three variables are introduced into the system:<br>• Size of the primary tumor and whether it has invaded nearby tissue (described as T0, T1, T2, etc)<br>• Regional lymph nodes that are involved (indicated as N0, N1, N2, etc);<br>• Metastasis (M0 or M1)<br>According to the selection of these 3 categories/variables, the application indicates the disease's stage of development (cancer extent), according to the above mentioned guideline.<br>According to the manufacturer, this information software simply facilitates the search and use of an international guideline that physicians usually consult via electronic file or paper format.
**Outcome**	The software does not perform an action on data other than simple search function, as per in MEDDEV Guidance 2.1/6.<br>The product does not therefore fulfill the definition of medical device, according to Directive 93/42/EEC, and should not be qualified as such.

EU, European Union; TNM, Classification of Malignant Tumors; UICC, International Union Against Cancer.

3 References

1. 21st Centruy Cures Act of 2016. (2016). HR 34. Section 3060. 114th Congress. https://www.congress.gov/114/plaws/publ255/PLAW-114publ255.pdf [Accessed May 18, 2022].

2. US Food and Drug Administration. (2022). Clinical Decision Support Software: Draft Guidance for Industry and Food and Drug Administration Staff. https://www.fda.gov/media/109618/download [Accessed May 18, 2022].

3. European Union. (2017). Regulation (EU) 2017/745 of the European Parliament and of the Council on Medical Devices. https://eur-lex.europa.eu/legal-content/EN/TXT/?uri=CELEX%3A32017R0745&qid=1652899471647 [Accessed May 18, 2022].

4. Medical Device Coordination Group. (2019). MDCG 2019-11 Guidance on Qualification and Classification of Software in Regulation (EU) 2017/745 - MDR and Regulation (EU) 2017/746 – IVDR. https://ec.europa.eu/health/system/files/2020-09/md_mdcg_2019_11_guidance_qualification_classification_software_en_0.pdf [Accessed May 18, 2022].

5. European Commission. Manual on Borderline and Classification in the Community Regulatory Framework for Medical Devices. Version 1.22 2019. https://ec.europa.eu/health/system/files/2020-08/md_borderline_manual_05_2019_en_0.pdf [Accessed May 18, 2022].
